# Using a genetic algorithm to derive a highly predictive and context-specific frailty index

**DOI:** 10.18632/aging.103118

**Published:** 2020-04-28

**Authors:** Alberto Zucchelli, Alessandra Marengoni, Debora Rizzuto, Amaia Calderón-Larrañaga, Maurizio Zucchelli, Roberto Bernabei, Graziano Onder, Laura Fratiglioni, Davide Liborio Vetrano

**Affiliations:** 1 Aging Research Center, Department of Neurobiology, Care sciences and Society, Karolinska Institutet, Stockholm 17165, Sweden; 2 Department of Information Engineering, University of Brescia, Brescia 25123, Italy; 3 Department of Clinical and Experimental Sciences, University of Brescia, Brescia 25123, Italy; 4 Stockholm Gerontology Research Center, Aldrecentrum, Stockholm 11346, Sweden; 5 Kandou Bus SA, Lausanne 1015, Switzerland; 6 Department of Geriatrics, Fondazione Policlinico “A. Gemelli” IRCCS and Catholic University of Rome, Rome 00168, Italy; 7 Department of Cardiovascular, Endocrine-metabolic Diseases and Aging, Istituto Superiore di Sanità, Rome 00161, Italy

**Keywords:** frailty, frailty index, genetic algorithm, geriatric

## Abstract

The frailty index (FI) is one of the most widespread tools used to predict poor, health-related outcomes in older persons. The selection of clinical and functional deficits to include in a FI is mostly based on the users’ clinical experience. However, this approach may not be sufficiently accurate to predict health outcomes in particular subgroups of individuals. In this study, we implemented an optimization algorithm, the *genetic algorithm*, to create a highly performant (FI) based on our prediction goals, rather than on a predetermined clinical selection of deficits, using data from the Swedish National Study on Aging and Care in Kungsholmen (SNAC-K) and 109 potential deficits identified in the dataset. The algorithm was personalized to obtain a FI with high discrimination ability in the prediction of mortality. The resulting FI included 40 deficits and showed areas under the curve consistently higher than 0.80 (range 0.81-0.90) in the prediction of 3-year and 6-year mortality in the whole sample and in sex and age subgroups. This methodology represents a promising opportunity to optimize the exploitation of medical and administrative databases in the construction of clinically relevant frailty indices.

## INTRODUCTION

With age, an individual’s likelihood of experiencing chronic disorders, loss of functionality, cognitive problems, and of dying increases. Similarly, the ability to recover from acute events, such as a hip fracture or pneumonia, decreases or is even absent [1, 2]. This resilience reduction is due to the progressive accumulation of biological deficits across different organs and systems, which progressively leads to a loss of homeostasis, eventually failing to support the organism [3, 4]. The concept of frailty has been proposed to measure such a process in clinical and research settings [5, 6].

During the last two decades, a number of different clinical operationalizations of frailty have been developed, among which is the frailty index (FI) [5, 7]. The FI is a powerful measure of biological age that, through the assessment of clinically observable health deficits (e.g. diseases, signs, symptoms, poor physical and cognitive function etc.), attempts to capture the biological entropic burden proper to each aging individual [8, 9], easing risk evaluation and prediction of negative health-outcomes (e.g. death, hospitalizations, falls, and post-procedure adverse events [10–17]). A high number of FIs have been built across different patient populations, settings and countries [10, 18]. During the last years, several other medical specialties embraced the concept of frailty, resulting in frequent implementation of FIs in daily clinical practice. For example, the FI is currently used to assess the risk to a patient’s before invasive procedures, to choose a given chemotherapy, or for prognostic evaluation in particular subsets of patients [19–24]. There are also examples of FIs adopted on a large scale, as is the case of the electronic FI used in UK primary care, which recently revolutionized the decision-making process of British general practitioners [25].

A FI is calculated as the ratio between the number of clinical deficits presented by an individual and the overall number of deficits taken into consideration [26]. Although seminal papers [7, 26, 27] offered a set of rules to identify deficits among the available variables, an objective procedure to select the deficits to include in a FI is lacking. For this reason, currently available FIs are mainly built based on the clinical experience of their users. However, this approach may not be sufficiently accurate to predict health outcomes in particular subgroups of individuals. For example, we previously showed that a clinical FI loses a significant part of its discriminative capacity in predicting mortality and hospitalizations when applied to younger individuals (i.e. <80 years old), probably due to scarcity of deficits discriminating health status at younger ages [17]. In this regard, an objective deficit selection based on the target outcome, instead of on clinical expertise, would allow the building of highly reliable data-driven FIs.

With this study, we propose the application of an optimization algorithm—the *genetic algorithm*, a well-known tool employed to solve optimization problems [28]— to create a FI based on our prediction goals, rather than on a predetermined clinical selection of deficits. *Genetic algorithms* have been extensively employed to find near-optimal solutions to complex problems in the presence of a non-manageable number of possible alternatives [29]. For the aim of this study, we personalized a *genetic algorithm* to create a FI for mortality prediction in older people, characterized by a high prognostic accuracy both in the whole population and in sex and age subsamples. We used data from the population-based Swedish National Study on Aging and Care in Kungsholmen. In this article, we provide proof of concept, namely, that the FI built through a *genetic algorithm* exhibits a higher predictive performance—stable across subsamples and time windows—than that shown by a FI whose deficits were clinically selected.

## RESULTS

### Implementation of the *genetic algorithm*

**Figure 1:**
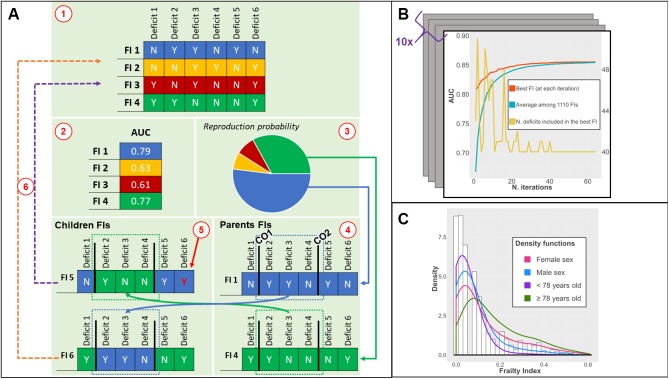
(**A**) Phases of the *genetic algorithm*: 1) an *initial population* of FIs is created; 2) the *fitness* (AUC) of each FI is tested; 3) the fittest FIs have higher chances to be selected for *recombination*; 4) two *crossing-over points* are randomly found for each *parent* FI: *children* FIs are created by combining different parts of *parents* FI; 5) a low probability of random *mutations* of a deficit is introduced; 6) *children* FIs replace the least fit FI; (**B**) Output of the *genetic algorithm*: iteration by iteration, the AUC of the best FI and average AUC of the population of FIs increases until convergence. The number of deficits included can vary iteration by iteration; (**C**) Distribution of the ga-FI in the whole population (histogram) and density functions in different subsamples. Abbreviations: FI = Frailty Index, AUC = Area under the Curve, CO = Crossing Over point; ga-FI = best *genetic algorithm*-derived FI.

A *genetic algorithm* is an optimization algorithm inspired by Darwin’s theory of evolution; it is employed to identify solutions to computationally complex problems through the principle of the “survival of the fittest” [28]. We implemented a *genetic algorithm* (Figure 1A) that, starting from 1100 randomly generated FIs, using the areas under the curve (AUC) as a metric of fitness assessment, and after a sufficient number of iterations (Figure 1B), identified a near-optimal FI (see Methods section for details). Each FI generated and tested by the GA was obtained as described in previous studies [7, 26]: the GA calculated the ratio between the number of deficits exhibited by each person and the total number of deficits chosen among those available (more details in the Methods section and Supplementary Table 3). The GA did not transform the deficits or assign any weights to the deficits included in the FIs. The data used for this study were obtained from the Swedish National Study on Aging and Care in Kungsholmen (SNAC-K), an ongoing population-based study started in 2001. The baseline characteristics of the study sample, randomly selected from the general population, are described in Table 1. The average age was 74.7 years (SD 11.2); 64.9% were female (N = 2182). Less than 6% of the participants were living in an institution and the prevalence of disability was 10%. All participants were clinically examined by physicians and nurses.

**Table 1:** Baseline characteristics of the SNAC-K population in the whole dataset, and in the training and test samples.

**Characteristics, N (%)**	**Total N = 3363**	**Training sample N = 2354 (70%)**	**Test sample N = 1009 (30%)**	**p**
*Demographic*				
**Age, mean (SD)**	74.7 (11.2)	74.9 (11.2)	74.3 (11.1)	0.217
**Female sex**	2182 (64.9%)	1532 (65.1%)	650 (64.4%)	0.713
**Living in institution**	191 (5.7%)	138 (5.9%)	53 (5.2%)	0.484
*Chronic diseases*				
**≥2 chronic conditions**	2900 (86.2%)	2019 (85.8%)	881 (87.3%)	0.233
**Atrial fibrillation**	324 (9.6%)	229 (9.7%)	95 (9.4%)	0.778
**Heart failure**	353 (10.5%)	245 (10.4%)	108 (10.7%)	0.798
**Ischemic heart disease**	514 (15.3%)	374 (15.9%)	140 (13.9%)	0.137
**COPD**	167 (5%)	108 (4.6%)	59 (5.8%)	0.123
**Dementia**	322 (9.6%)	234 (9.9%)	88 (8.7%)	0.271
**Solid neoplasms**	299 (8.9%)	217 (9.2%)	82 (8.1%)	0.308
*Cognitive and physical function*				
**Walking speed ≤ 0.8 m/s**	834 (26.9%)	592 (27.3%)	242 (25.9%)	0.447
**≥1 ADL impaired**	327 (9.7%)	232 (9.9%)	95 (9.4%)	0.693
**≥1 IADL impaired**	3195 (95%)	2241 (95.2%)	954 (94.5%)	0.427
**MMSE ≤ 27**	462 (14.7%)	318 (14.5%)	144 (15.0%)	0.690
*Outcomes*				
**3-year mortality**	477 (14.2%)	347 (14.7%)	130 (12.9%)	0.157
**6-year mortality**	927 (27.6%)	661 (28.1%)	266 (26.4%)	0.307

Abbreviations: SD = standard deviation; COPD = Chronic Obstructive Pulmonary Disease; m/s = meters per second; ADL = Activities of Daily Living; IADL = Instrumental Activities of Daily Living; MMSE = Mini Mental State Examination.

Missing data: 329 for BMI, 258 for walking speed, 213 for MMSE.

We applied the *genetic algorithm* in a random sample of our data (i.e. training sample, 70%, N = 2354). The resulting FI was described and compared with a clinical FI in the remaining 30% (i.e. test sample, N = 1009) (Supplementary Table 1). From the test sample, 163 (16.1%) participants died during the first three years and almost 30% (N = 297) during the first six years (Table 1). We ran the *genetic algorithm* (Figure 1A) in the training sample 10 times, obtaining 10 near-optimal FIs. On average, it took 59 iterations (range 45-63) to reach convergence (i.e. a point beyond which the algorithm is unlikely to find better solutions). The resulting FIs showed similar discriminant capacity (average AUC in the training sample = 0.854; SD = 0.02) and low overfitting (range of differences in AUC when applied to test sample: -0.05 to +0.04).

### Characteristics of the near-optimal frailty index

The FI showing the highest accuracy in the training sample across the whole population and in the different subsamples was chosen for the analyses on the test sample. The best *genetic algorithm*-derived FI (ga-FI) was obtained after 63 iterations (Figure 1B) and included 40 deficits (Supplementary Table 3) that explore different domains of health. Nineteen were chronic diseases; 13 referred to function and physical performance measures; four were related to socio-economic status; one was a measure of cognitive performance (i.e. Mini Mental State Examination); one was a self-reported problem (i.e. loss of appetite); one was a sign (i.e. abnormal patellar reflex); and one was a measure of healthcare utilization (i.e. at least one hospitalization in the previous year). In the test sample, the ga-FI had 17 missing values (2%). The ga-FI exhibited a γ-distribution (Figure 1C) and a maximum value lower than 0.70 (i.e. 0.60); both these characteristics have been reported in the literature as depicting reliable FIs [7, 26, 30, 31]. The skewness of the distribution resulted more positive in participants <78 years old and more negative in the oldest group. No distribution differences arose between males and females.

### Performance of the derived frailty index

**Figure 2:**
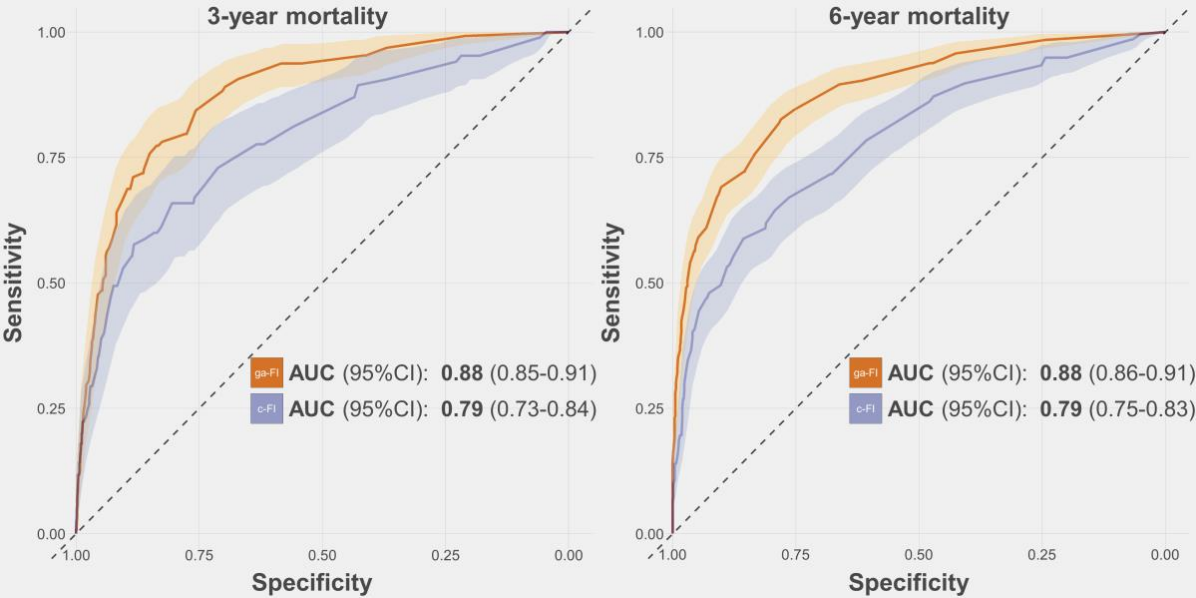
**Receiver-Operating-Characteristics Curve and Areas Under the Curve (AUC) for the prediction of 3-year and 6-year mortality obtained for the ga-FI and c-FI in the test sample.** Abbreviations: 95% CI = 95% confidence intervals; ga-FI = best *genetic algorithm*-derived Frailty Index; c-FI = clinically generated Frailty Index.

**Figure 3:**
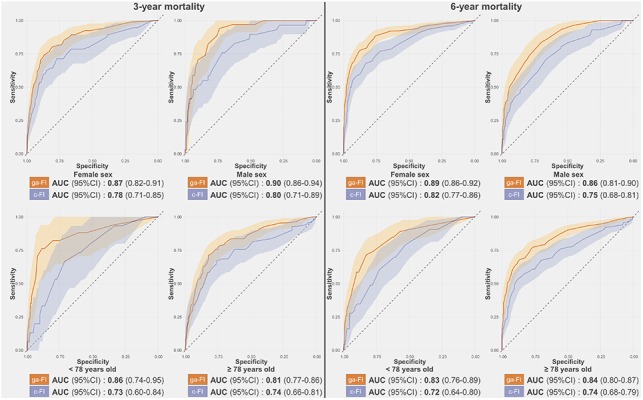
**Receiver-Operating-Characteristics Curve and Areas Under the Curve (AUC) for the prediction of 3-year and 6-year mortality obtained for the ga-FI and c-FI in the test sample, in different subsamples.** Abbreviations: 95% CI = 95% confidence intervals; ga-FI = best *genetic algorithm*-derived Frailty Index; c-FI = clinically generated Frailty Index.

The ga-FI exhibited AUCs consistently higher than 0.80 across the whole test sample and its age and sex subsamples, both for 3-year and 6-year mortality prediction. Namely, the AUC for 3-year and 6-year mortality was 0.88 (95% confidence interval [CI] 0.85-0.91) and 0.88 (95% CI 0.86-0.91), respectively (Figure 2 and Supplementary Table 2). When applied to the subsamples, the ga-FI AUCs ranged from 0.81 (3-year mortality, older individuals) to 0.90 (3-year mortality, male sex). In a multivariate logistic regression adjusted by age, sex, cognitive status, and walking speed < 0.8 m/s, each increase by 0.1 in the ga-FI resulted in a 3-year mortality odds ratio of 2.17 (95% CI 1.54-3.04) and in a 6-year mortality odds ratio of 3.88 (95% CI 2.63-5.74). Comparing the ga-FI with a FI we had previously built on the basis of our clinical expertise (c-FI), which included 45 deficits [17], we found that only about half (N = 24) of the deficits were shared by the two indices (Supplementary Table 3). As depicted in Figures 2 and 3, in the test sample, the ga-FI showed higher areas under the curve (AUCs) in the prediction of mortality in the whole population and in all subsamples, when compared with the c-FI. Differences between the AUCs exhibited by the ga-FI and c-FI did not reach statistical significance only in the younger and older subsamples for 3-year mortality (Supplementary Table 2).

### Simulation study of randomly generated frailty indices

**Figure 4:**
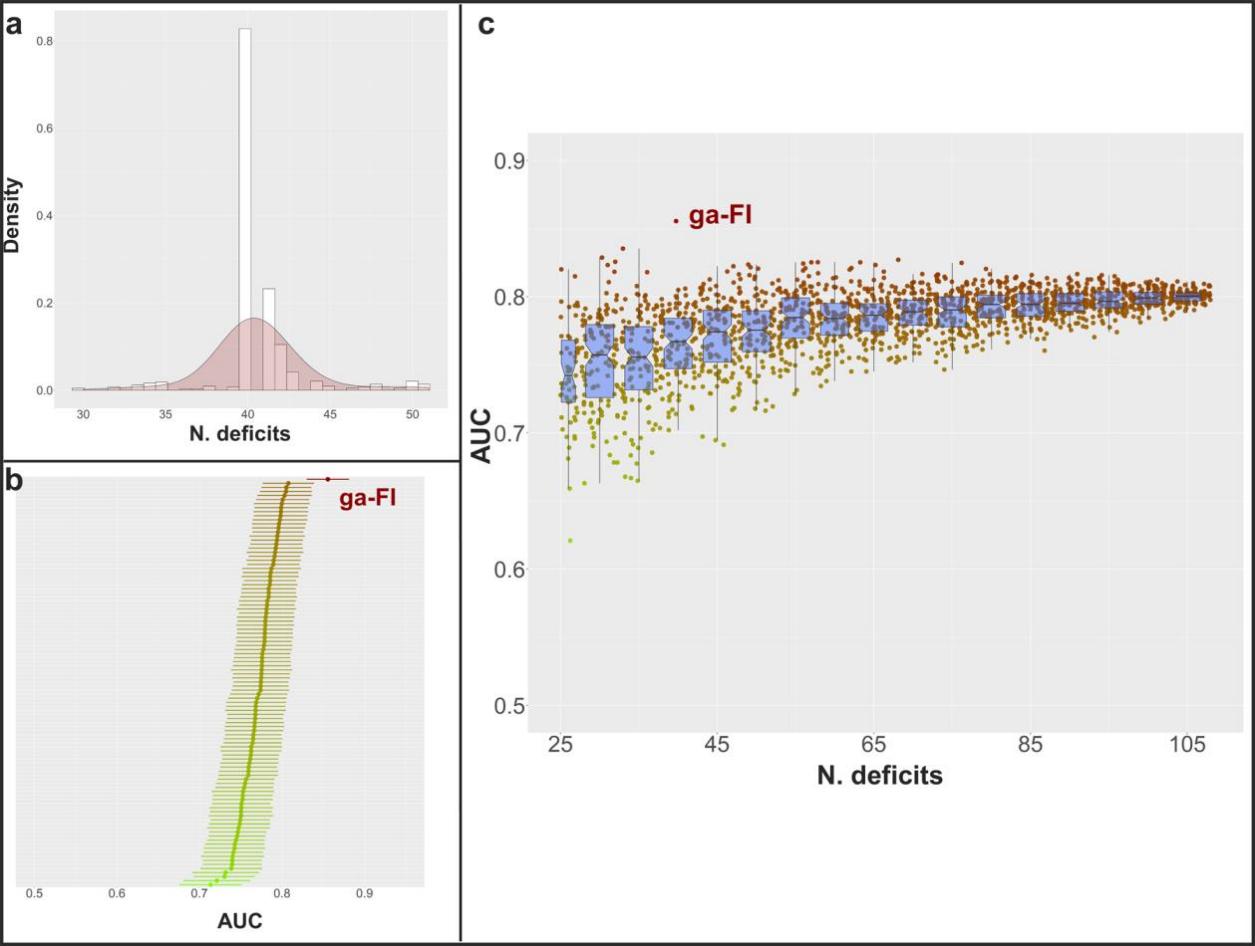
(**A**) Distribution of the number of selected deficits for the best FIs in each iteration among the 10 *genetic algorithm* cycles; (**B**) mean AUC and 95% confidence intervals in the prediction of 3-year and 6-year mortality in the whole population and in sex- and age subgroups (calculated in the complete dataset) for 100 randomly generated FIs including 40 deficits (mean AUC for ga-FI, calculated in the complete dataset, shown in red); (**C**) mean AUC in the prediction of 3-year and 6-year mortality in the whole population and in sex- and age subgroups (calculated in the complete dataset) for more than 2000 randomly generated FIs including 25-108 deficits (mean AUC for ga-FI, calculated in the complete dataset, shown in red) – boxplots show median and 2^nd^ and 3^rd^ quartiles of mean AUC for FIs with similar number of deficits.

As shown in Figure 4A, almost all the best FIs from each iteration over ten runs of the *genetic algorithm* included 40 deficits. In order to investigate the meaning of such a finding, we randomly generated 100 FIs that included 40 deficits, and compared their AUCs with the ga-FI. As shown in Figure 4B, none of the randomly generated FIs reached the same predictive accuracy of the ga-FI. Finally, upon generating about 2000 random FIs that included between 25 and 108 deficits, none of them presented with an AUC as high as that of the ga-FI (Figure 4C).

## DISCUSSION

In the present study, we showed that an optimization algorithm can be used to guide the selection into which and how many deficits are to be included in a highly performant FI. To the best of our knowledge, this is the first time robust and standardized optimization methodology has been employed for this purpose. The adoption of the *genetic algorithm* led to a FI exhibiting AUCs higher than most of those reported in previous literature for the prediction of mortality, stable across different age and sex subgroups, and both in the short and long term [25, 32–34].

In recent decades, several authors adopted the FI as a tool to quantify the risk of developing poor, health-related outcomes in different settings and among persons affected by different diseases. Such proliferation may be explained by the fact that several studies showed that once a minimum number of deficits has been included in a FI, it is strongly associated with poor outcomes, irrespective of the list of variables included [5, 8, 26, 35]. Our findings confirm that the strong relationship between deficit accumulation (operationalized as a FI) and survival tolerates a considerable heterogeneity in the group of variables taken into consideration. Nevertheless, our study shows that the quantitative and qualitative aspects of deficit selection significantly impact the discriminative performance of the resulting FI.

### How many deficits?

The inclusion of a minimum of 30-40 deficits has been recommended to obtain a reliable FI. However, it has been suggested that a higher number of variables may improve the precision of a FI’s estimates [26]. While aiming for an accurate FI, we found that the *genetic algorithm* included 40 deficits in the fittest FI in our dataset, and that FIs including more or less deficits had a higher probability to be discarded by the *genetic algorithm*. Indeed, almost all the best FIs (in each iteration over ten runs of the *genetic algorithm*) included 40 deficits, even if no limitations in the number of deficits (after the first iteration) were introduced. However, upon generating 2000 random FIs, which included between 25 and 108 deficits, we observed that the median AUC increased up to within 65-70 deficits, and then plateaued. Arguably, the inclusion of too few variables may lead to a less precise FI, more sensible to missing values. At the same time, our simulation suggests that the inclusion of too many deficits would not translate into a higher discriminant capacity; indeed, this is likely to generate statistical noise, limiting the further improvement of the FI. Lin S. and colleagues [34] showed that a 139-deficit and a 35-deficit FI exhibited similar AUCs in the prediction of mortality. Interestingly, the 35 deficits were identified using an information reduction technique, i.e. exploratory factor analysis. Furthermore, in a recent study based on data from the European Prospective Investigation into Cancer and Nutrition (EPIC) [36], a multi-collinearity analysis allowed the authors to discard seven deficits that probably did not add any information to a 39-deficit FI. Yet, we cannot conclude that there is an optimal number of deficits to be included in a FI in absolute terms. Rather, it is likely there is an optimal number of deficits to build a FI for each given population with a given assortment of potential deficits. Furthermore, our findings suggest that the selection of deficits is particularly important when the total number of deficits included is low. This is evident from the scattered AUCs from randomly generated FIs with a low number of deficits. In this regard, the *genetic algorithm* represents promising methodology for the creation of highly-performing FIs in different settings and datasets, without *a-priori* knowledge about the optimal number of deficits to be included.

### Which deficits?

Clinicians and researchers build their FIs selecting the deficits on the basis of their clinical experience, relying on the recommendations provided by Searle SD et al. [26] In accordance with these recommendations, a deficit should 1) be associated with health status, 2) have a prevalence that generally increases with age, and 3) not saturate too early with age. Moreover, the complete list of deficits should cover different domains of health. Our *genetic algorithm* was implemented to be blinded to these recommendations and to deficit distribution in the population. Thirty-seven out of the 40 deficits included in the ga-FI were fully compliant with the aforementioned recommendations, while three deficits presented a similar prevalence among younger and older individuals. Among the 109 deficits we selected to run the *genetic algorithm*, 80% (N = 87) presented a prevalence among older individuals higher than that exhibited among younger ones, and none exhibited a risk of premature saturation; all these deficits could, in theory, be included in the resulting FI. However, the ga-FI included only 40 deficits and, interestingly, the *genetic algorithm* excluded several deficits exhibiting well-known associations with aging, mortality, and frailty (such as ischaemic heart disease, thyroid diseases, osteoporosis, diabetes, chronic kidney disease, hypertension, self-reported low quality of life, some self-reported symptoms, etc.). These deficits are typically included in the FIs proposed in the literature [7, 10, 17, 18, 25, 36]. In spite of such exclusions, the ga-FI showed a higher AUC (0.88 in the whole sample) for mortality than those reported in the literature (ranging between 0.64 and 0.80 [25, 32–34, 37]), and a stronger association with mortality than those reported in a recent meta-analysis [10]. Indeed, the odds ratios per 0.1 FI increase from the meta-analysis range, between 1.05 and 1.73, whereas the corresponding figure for our ga-FI was 3.88. It is likely that, as reported in the previous paragraph, different deficits convey similar information to the FI, and their indiscriminate inclusion increases statistical noise. For example, although ischaemic heart disease was excluded from the ga-FI, heart failure (a common consequence of myocardial infraction) was included; although heart failure is less prevalent than myocardial infarction (Supplementary Table 3), its association with mortality is stronger (data not shown). While minimizing the number of deficits, the *genetic algorithm* maximizes the information included in a FI, which is likely to increase the FI’s discriminant capacity in mortality prediction.

The lowest difference in the AUCs between the ga-FI and the c-FI was found in the prediction of 3-year mortality among older individuals, where 24 deficits were shared between these two FIs. These findings corroborate the idea that the clinical selection of deficits may lead to the construction of an accurate FI in specific subsamples. However, the *genetic algorithm* provided a FI characterized by a consistently higher discriminative capacity in different subsamples using the same 40 deficits.

### Strengths and limitations

Box 1 shows the main advantages offered by the implementation of a *genetic algorithm* in the construction of a FI. G*enetic algorithms* can be highly personalized according to researchers’ or clinicians’ aims and needs—both the characteristics of the generated FI and the *fitness* criteria can be adapted to the goals of the users developing it (see methods for further details). *Genetic algorithms* were first proposed several decades ago [28] and are still commonly used due to the convenience of their adoption [38–42]. Several papers are available for researchers and physicians to understand the limits, possibilities and methodology used in *genetic algorithm*s [43, 44]. Indeed, *genetic algorithms* may be implemented in different settings, using commonly employed statistical software. Furthermore, *genetic algorithms*, although strongly related to random numbers (see methods for further details), propose solutions according to user pre-defined criteria. Researchers can still infer about the resulting solutions—this is not always possible using high accuracy “black-box” methods (such as neural networks) [45]. In spite of these advantages, the use of the *genetic algorithm* in our study should be understood, bearing certain limitations in mind. Firstly, *genetic algorithms* (as other algorithms) tend to overfit. We limited overfitting by employing two separate subsamples (a training and test sample), and we presented the results through bootstrapping techniques. Nevertheless further studies are needed to inspect the predictive accuracy of the ga-FI in different datasets, such as follow-up waves of SNAC-K. Secondly, we tested the *genetic algorithm* for the computation of an optimized FI using only SNAC-K data. These data are gathered through a set of interviews, tests and examinations performed by trained nurses, physicians and neuropsychologists, which guarantees more exhaustive and comprehensive data collection compared to automatically generated administrative data. Further studies are needed to understand the optimization power a *genetic algorithm* has on data coming from different settings. Lastly, it is possible that the parameters presented in this paper (such as initial population size, mutation probability, elitism—see methods for details) allowed us to obtain a good accuracy-to-computing speed ratio in SNAC-K, but may require further testing in other datasets.

### Relevance and future applications

In this study, we showed that a *genetic algorithm* may be helpful in selecting those deficits responsible for an optimal predictive capacity, whether it is in the general population or in specific subsamples. The *genetic algorithm* might be used to minimize the number of deficits needed to obtain a reliable FI in different subsamples; this may strongly contribute to cost reductions when a FI is used to stratify risk in a large population, for example. *Genetic algorithm*s can be personalized with different *fitness functions*; in other words, it would be possible to select other outcomes (such as hospitalizations, healthcare resource use, falls, etc.) to adapt the FI to their prediction. Furthermore, the *genetic algorithm* could be used to create a highly accurate FI in a particular subsample, for example, patients undergoing specific surgical procedures or groups affected by the same index disease (e.g. HIV). Additionally, the metrics used to assess *fitness* can also be modified—a FI with higher sensitivity might be used for screening purposes, for example. *Genetic algorithms* are well known algorithms, although seldom used in medical research. In consideration of the increasing number of available large databases [46], these algorithms might help to select features of interest without having to rely on black-box methodologies [45]. Interestingly, a previous study [47] showed how non-linear modelling (obtained using machine learning techniques) may help to increase the accuracy in the prediction of mortality, compared to the simple deficit ratio calculation. To note, the *genetic algorithm* may be employed to find the most suitable weights for the deficits to be included in a non-linear form of FI, in order to increase its accuracy.

## CONCLUSIONS

The frailty index is a recognized clinically relevant tool to support medical decisions in the treatment and care of older adults. In this study, we present the implementation of an algorithm that allows to overcome the limitations posed by feature selection, based mostly on clinicians’ expertise, which may lead to wrong or biased results. We show that the *genetic algorithm* generates a FI with a consistently high accuracy in the prediction of mortality in different subsamples, obtaining higher AUCs than the ones exhibited by a FI whose deficits were selected based on our own expertise. This methodology represents a promising opportunity to optimize the exploitation of clinical and administrative databases in the construction of highly performant frailty indices.

Box 1 **Advantages offered by the *genetic algorithm* in the optimization of a frailty index.**

*Genetic algorithms’* strength-points:

1. Is user-focused: the main characteristics of candidate solutions can be specified by the user.
2. Is goal-directed: the definition of “goodness” of a solution is defined by the user.
3. Is data-driven: it might be used where clinical experience is insufficient but data are available.
4. Is transparent: although largely based on random numbers, the selection of solutions is user-determined. In contrast with black-box methods (as most machine-learning algorithms), inference about the selected solution is possible.

## MATERIALS AND METHODS

### Study population

Data were obtained from the Swedish National Study on Aging and Care in Kungsholmen (SNAC-K), an ongoing population-based study started in 2001. Persons aged at least 60 years old and living in a central area of Stockholm (Sweden), either at home or in institutions, were asked to participate in the study. Standard questionnaires, interviews, medical examinations, instrumental evaluations, as well as physical and neuropsychological tests were employed to retrieve information for the 3363 individuals enrolled in the study. Further details are available in a previous study [48]. The Regional Ethical Review Board of Stockholm approved every phase of SNAC-K. Each participant, or their proxies in case of cognitive decline, signed a written informed consent form. The study population was randomly split into two samples: 70% (N = 2354) was used to run the algorithm (i.e. training sample), while the remaining 30% (N = 1009) was employed to describe the resulting FI and for the comparison with a clinical FI (i.e. test sample).

### Identification of potential deficits

A variable was considered to be a potential deficit if it was likely associated with individuals’ health status. For the aim of the present study, we decided not to limit the selection of potential deficits to those whose prevalence grew with age, as suggested by Searle SD [26]. Our hypothesis was in fact that deficits whose prevalence varied with age (i.e. increasing, with bimodal pattern, or even decreasing) may be able to add meaningful information regarding health. We identified a total of 109 potential deficits; these variables were re-codified into dichotomous deficits in order to be suitable for FI creation [49]. Cut-offs used for dichotomization were based on the literature and are shown in Supplementary Table 3. The total number of possible FIs (including between 30 and 70 deficits and based on 109 variables identified) was higher than 6.4×10^32^.

### The *genetic algorithm*

*A genetic algorithm* starts from a limited number of randomly generated solutions and applies a *selective pressure*. Solutions more adherent to pre-defined criteria (i.e. *the fittest individuals*) have higher chances to be combined with each other, generating new solutions. These newly generated solutions (i.e. children solutions), likely incorporating *parents*’ characteristics responsible for the increased *fitness*, replace the less fit solutions from the previous iteration. Iteration by iteration, the algorithm produces solutions that are increasingly adherent to the pre-defined criteria set by the investigators.

Figure 1A illustrates the six phases coded in our *genetic algorithm*.

1. *Creation of the initial set of solutions:* in this phase, the *genetic algorithm* randomly creates a set of solutions (*initial population*, in *genetic algorithm* terminology). These solutions represent the starting point used by the *genetic algorithm* to search (and create) more fit solutions to the problem. Our *genetic algorithm* created 1100 FIs. We set the initial number of FI to 10 times the number of potential deficits. After different trials, we found that this number offered a good balance between late convergence and time of execution. Each potential deficit had a uniform probability to be included (or excluded) from each FI. Each randomly created FI was represented by a vector (*chromosome* in *genetic algorithm* terminology) with dimension 109 (total number of potential deficits found in SNAC-K): a 1 or 0 (*allele* in *genetic algorithm* terminology) was recorded for each vector component, according to the status of the deficit (included or excluded, respectively). In this phase, the number of deficits included (i.e. the number of “1”s recorded) in each FI ranged between 30 and 70. To note, we did not introduce any constraints on the number of deficits to be considered in the next phases of the algorithm. A FI was built from each vector; the sum of the deficits included was divided by the total number of non-missing deficits taken into consideration. If the number of missing deficits exceeded 10% of those included in the FI, a missing value for the FI was registered. If the total number of missing values for the FI was higher than 177 (7.5% of the training sample), the FI was excluded.
2. *Fitness evaluation:* in this phase, the *genetic algorithm* assesses the *fitness* of each solution according to the definition of *goodness* set by the developing team. Our *genetic algorithm* was coded to find the most accurate FI for the prediction of mortality in the whole study population and different subsamples. The accuracy was assessed calculating the mathematical mean between the Areas Under the Receiver-Operating-Characteristic (AUC) in the prediction of 3-year and 6-year mortality, exhibited in the whole study sample and in four subsamples (i.e. males and females, individuals younger and older than 78 years—the median age in SNAC-K). The AUCs were obtained from non-parametric ROC analysis.
3. *Selection:* in this phase, the *genetic algorithm* selects the *parent* solutions (i.e. the fittest solutions out of the 1100) to be re-combined in new solutions, likely increasing the general fitness of the *population* of solutions. We employed a *proportionate fitness selection operator* for this. Shortly, the probability of selection for each FI was obtained according to the following formula:
  $$p_{i}=\;\frac{\bar{AUC_{i}}}{\sum_{j=1}^{1100}\;\bar{AUC_{j}}}$$
  where *i* is the considered FI and $\bar{AUC}$ is the mean AUC obtained in the *fitness evaluation* phase. This method allows the increase of the probability in selecting the most accurate FIs (although it is never equal to 1—they are not necessarily selected) and to decrease the probability in selecting those FIs with a lower accuracy (although it is never equal to 0—they are not necessarily excluded). This method is also known as “roulette wheel selection”. Similar to the casino game, where a ball is used to determine the winning number among all possible numbers, a random probability *p_i_* is used to select candidate solutions for re-combination. The probability for a FI to be selected is proportional to its accuracy (conversely, in the casino game, the pocket in the roulette wheel in which the ball may stop is equally probable for all numbers). We obtained 1067 couples of FIs for the *re-combination* phase: the 33 most accurate FIs in every iteration were maintained for the subsequent iteration (*elitism* = 3%). There were no constraints on the number of times a FI could be selected and the same FI could also be selected for both elements of the couple (therefore, the recombination phase would not have any effect).
4. *Recombination (crossover)*: in this phase, the *genetic algorithm* re-combines the selected solutions (*parents*) to generate *children* solutions (*offspring* in *genetic algorithm* terminology). Each *child* solution includes sections of the *parents’* *chromosome*. In our study, two random numbers were generated for each couple of selected FIs. These numbers, called *crossing-over points* in *genetic algorithm* terminology, identified the “switching” positions in the vectors representing the FIs. A new vector (i.e. a new FI) was created by combining the components between the first component and the component identified by the first random number of the first vector, the components between the positions identified by the first and the second random numbers of the second vector, and the components between the second random number and the last component of the first vector again (Figure 1A). This procedure was repeated for all the couples selected.
5. *Mutation*: in this phase, the *genetic algorithm* has a low but existing probability to randomly change part of a *chromosome*. This phase allows the *genetic algorithm* to find new solutions that in theory cannot be generated starting from the *initial population*. On the other side, high *mutation* *rates* can possibly change solutions with high *fitness*. In our *genetic algorithm*, we established a 3% chance that a random component of a vector was changed (therefore excluding or including a random deficits).
6. *Population replacement and termination:* in this phase, the *genetic algorithm* replaces the solutions included in the *initial population* with those generated in the *re-combination phase*. In our *genetic algorithm*, re-combined FIs replaced the 97% least accurate FIs in the *initial population* (see point 3). The *genetic algorithm* ran until at least one of the following conditions was met: 1) 150 iterations were reached, or 2) the average AUC of the best FI did not show any AUC increase for seven consecutive iterations.

We run the *genetic algorithm* 10 times, therefore starting from 10 different *initial populations*, on the same training sample. The FI showing the highest average AUC most frequently across the whole population and different subsamples in the training sample was considered our best ga-FI, and was compared head-to-head with the c-FI (see below). Figure 1B summarizes the cycle that led to the best ga-FI.

### The clinical Frailty Index (c-FI.)

The c-FI was created based on a previous published work [17]. It includes 45 deficits, clinically selected by two geriatricians (Supplementary Table 3). It showed AUCs similar to other well-known geriatric indicators (such as walking speed) in the prediction of mortality and hospitalizations. In the test sample, the number of missing values was 96 (9%).

### Statistical analyses

Differences in the characteristics of the study population and its subsamples were described using means and standard deviations or absolute numbers and proportions and investigated using t-tests and chi-squared tests, as appropriate. The performance of the ga-FI and the comparison with the c-FI were conducted in the test sample. AUCs, their confidence intervals, and their comparison were obtained through non-parametric ROC analyses, employing stratified bootstrapping replacement technique [50] (N = 2000). Confidence intervals for specificities at given sensitivities for the ROC curves were also obtained through stratified bootstrapping replacement technique (N = 2000). Panels b and c in Figure 4 were obtained by creating FIs that randomly included a given number of deficits: their AUCs were calculated using non-parametric ROC analyses in the complete dataset. The association between the ga-FI and mortality (3-year and 6-year) was tested running logistic regressions in the test sample, adjusted for age, sex, MMSE < 27, and walking speed ≤ 0.8 m/s. An α-level of 0.05 was used for all the analyses. The genetic algorithm was coded entirely in STATA 15 (Statacorp, Texas – USA). The analyses were conducted using STATA 15 and R 3.6.1 (R Foundation for Statistical Computing, Wien – Austria) [50–52].

### Ethics approval and consent to participate

Every wave of the study was approved by the Regional Ethical Review Board in Stockholm, Sweden. Written informed consent was obtained from each participant, or from a proxy, in case of cognitive impairment. Public or patients were not involved during the development of this study: anyhow, we plan to disseminate the findings of this research to participants of SNAC-K and to the public.

### Availability of data and materials

Data are from the SNAC-K project, a population-based study on aging and dementia (http://www.snac-k.se/). Access to these original data is available to the research community upon approval by the SNAC-K data management and maintenance committee. Applications for accessing these data can be submitted to Maria Wahlberg (Maria.Wahlberg@ki.se) at the Aging Research Center, Karolinska Institutet.

### Transparency statement

The lead author (AZ) affirm that the manuscript is an honest, accurate, and transparent account of the study being reported; that no important aspects of the study have been omitted; and that any discrepancies from the study as originally planned have been explained.

## Supplementary Material

Supplementary Tables 1, 2

Supplementary Table 3
