# Supplementary material for: Using a genetic algorithm to derive a highly predictive and context-specific frailty index
Source: Aging (Albany NY). 2020 Apr 28;12(8):7561–75. doi: 10.18632/aging.103118 (PMC7202492; doi:10.18632/aging.103118)
Supplement: Supplementary Tables 1, 2 [file aging-12-103118-s002..pdf]

SUPPLEMENTARY TABLES

Supplementary Table 1. Baseline characteristics of the SNAC-K population included in the validation sample by age and sex.

Characteristics, N (%)	Men	Women	< 78 Years old	≥ 78 Years old
	N = 359 (35.6%)	N = 650 (64.4%)	N = 559 (55.4%)	N = 450 (44.6%)
Demographic				
**Age, mean (SD)**	71.3 (10.2)	76.0 (11.2)	65.7 (4.8)	85.1 (6.0)
**Female sex**	-	650 (100.0%)	318 (56.9%)	332 (73.8%)
**Living in institution**	6 (1.7%)	47 (7.2%)	4 (0.7%)	49 (10.9%)
Chronic diseases				
**≥2 chronic conditions**	297 (82.7%)	584 (89.8%)	443 (79.2%)	438 (97.3%)
**Dementia**	14 (3.9%)	74 (11.4%)	10 (1.8%)	78 (17.3%)
**Solid neoplasms**	32 (8.9%)	50 (7.7%)	32 (5.7%)	50 (11.1%)
Cognitive and physical function				
**Walking speed ≤ 0.8 m/s**	49 (14.3%)	193 (32.7%)	37 (6.8%)	205 (52.8%)
**≥1 ADL impaired**	13 (3.6%)	82 (12.6%)	9 (1.6%)	86 (19.1%)
**≥1 IADL impaired**	341 (95.0%)	613 (94.3%)	526 (94.1%)	428 (95.1%)
**MMSE ≤ 27**	24 (7.0%)	120 (19.5%)	24 (4.4%)	120 (29.3%)
Outcomes				
**3-year mortality**	43 (12.0%)	87 (13.4%)	15 (2.7%)	115 (25.6%)
**6-year mortality**	80 (22.3%)	186 (28.6%)	47 (8.4%)	219 (48.7%)

Abbreviations: SD = standard deviation; COPD = Chronic Obstructive Pulmonary Disease; m/s = meters per second; ADL = Activities of Daily Living; IADL = Instrumental Activities of Daily Living; MMSE = MiniMental State Examination.
Missing: 88 for BMI, 76 for walking speed, 52 for MMSE.

Supplementary Table 2. Comparison between areas under the curves (AUC, 95% confidence intervals) in the prediction of different outcomes obtained for the ga-FI and c-FI.

	AUC	AUC	p
	Best ga-FI	c-FI	
**3-year mortality**	0.88 (0.85-0.91)	0.79 (0.73-0.84)	0.006
**3-year mortality - female**	0.87 (0.82-0.91)	0.78 (0.71-0.85)	0.004
**3-year mortality - male**	0.90 (0.86-0.94)	0.80 (0.71-0.89)	0.048
**3-year mortality - older**	0.81 (0.77-0.86)	0.74 (0.66-0.81)	0.067
**3-year mortality – younger**	0.86 (0.74-0.95)	0.73 (0.60-0.84)	0.108
**6-year mortality**	0.88 (0.86-0.91)	0.79 (0.75-0.83)	<0.001
**6-year mortality - female**	0.89 (0.86-0.92)	0.82 (0.77-0.86)	0.006
**6-year mortality - male**	0.86 (0.81-0.90)	0.75 (0.68-0.81)	0.007
**6-year mortality - older**	0.84 (0.80-0.87)	0.74 (0.68-0.79)	0.003
**6-year mortality - younger**	0.83 (0.76-0.89)	0.72 (0.64-0.80)	0.045

All data were obtained employing 2000 stratified bootstrap replicates.
