# Supplementary material for: Using a genetic algorithm to derive a highly predictive and context-specific frailty index
Source: Aging (Albany NY). 2020 Apr 28;12(8):7561–75. doi: 10.18632/aging.103118 (PMC7202492; doi:10.18632/aging.103118)
Supplement: Supplementary Table 3 [file aging-12-103118-s001..docx]

**Supplementary Table 3.** **List of 109 deficits found in SNAC-K.**

| DEFICIT | N (%) | Missing values | N (%)  < 78 years old | N (%)  ≥ 78 years old | p | Ga-FI | c-FI |
| --- | --- | --- | --- | --- | --- | --- | --- |
| Hypertension | 2277 (67.7%) | 0 | 1154 (64.8%) | 1123 (71%) | < 0.001 |  | **🗸** |
| Dyslipidemia | 1558 (46.3%) | 0 | 934 (52.4%) | 624 (39.5%) | < 0.001 |  |  |
| Reporting difficulties in climbing a flight of stairs | 1060 (45%) | 1007 | 438 (29.9%) | 622 (70%) | < 0.001 |  |  |
| Reporting any pain in the last 4 weeks | 1167 (36%) | 125 | 612 (34.7%) | 555 (37.6%) | 0.085 |  | **🗸** |
| Sedentary behaviour (engaging in physical activity less than 2-3 times/month) | 1163 (34.6%) | 0 | 374 (21%) | 789 (49.9%) | < 0.001 | **🗸** |  |
| Hand grip (lower than 20th percentile adjusted by sex and BMI) | 1128 (33.5%) | 0 | 469 (26.3%) | 659 (41.7%) | < 0.001 |  |  |
| Poor social network | 766 (33.4%) | 1068 | 392 (27.3%) | 374 (43.6%) | < 0.001 | **🗸** |  |
| Chronic Kidney Disease | 1117 (33.2%) | 0 | 248 (13.9%) | 869 (55%) | < 0.001 |  | **🗸** |
| Abnormal patellar reflex (at physical examination) | 1006 (31.3%) | 148 | 331 (19%) | 675 (45.8%) | < 0.001 | **🗸** |  |
| Feeling tired most of the time | 683 (29.2%) | 1021 | 271 (18.5%) | 412 (47%) | < 0.001 |  |  |
| Feeling older than actual age | 669 (28.6%) | 1023 | 450 (30.9%) | 219 (24.8%) | 0.002 |  | **🗸** |
| Walking speed lower than 0.8 m/s | 834 (26.9%) | 258 | 125 (7.2%) | 709 (51.9%) | < 0.001 | **🗸** |  |
| Being widowed | 896 (26.8%) | 15 | 147 (8.3%) | 749 (47.7%) | < 0.001 | **🗸** |  |
| Feeling lonely most of the time | 849 (26.3%) | 129 | 361 (20.6%) | 488 (33%) | < 0.001 |  |  |
| Abnormal balance in tandem position | 758 (25.6%) | 400 | 109 (6.3%) | 649 (52.2%) | < 0.001 |  |  |
| Reporting change in smell | 757 (24.2%) | 240 | 410 (24%) | 347 (24.6%) | 0.706 |  |  |
| Blue collar job (previous or current) | 792 (24.2%) | 84 | 291 (16.4%) | 501 (33.2%) | < 0.001 |  |  |
| Reporting sleeping difficulties | 789 (23.6%) | 13 | 386 (21.7%) | 403 (25.6%) | 0.008 |  |  |
| Using a stick as walking aid | 771 (23.1%) | 30 | 95 (5.4%) | 676 (43.3%) | < 0.001 | **🗸** | **🗸** |
| Reporting low quality of life | 428 (18.2%) | 1016 | 215 (14.7%) | 213 (24%) | < 0.001 |  | **🗸** |
| Anxiety | 574 (17.8%) | 143 | 299 (16.9%) | 275 (18.9%) | 0.135 |  | **🗸** |
| Elementary education | 590 (17.7%) | 32 | 153 (8.6%) | 437 (28.2%) | < 0.001 | **🗸** |  |
| Inability to shop alone | 588 (17.6%) | 28 | 49 (2.8%) | 539 (34.6%) | < 0.001 |  | **🗸** |
| At least one acute hospitalization in the previous year | 562 (16.7%) | 0 | 200 (11.2%) | 362 (22.9%) | < 0.001 | **🗸** |  |
| Inability to do the laundry alone | 542 (16.2%) | 27 | 42 (2.4%) | 500 (32.1%) | < 0.001 | **🗸** | **🗸** |
| Heavy alcohol consumption | 510 (15.6%) | 97 | 362 (20.5%) | 148 (9.9%) | < 0.001 |  |  |
| Exhibiting a hostile behaviour | 489 (15.3%) | 165 | 292 (16.6%) | 197 (13.7%) | 0.027 |  |  |
| Ischemic heart disease | 514 (15.3%) | 0 | 138 (7.7%) | 376 (23.8%) | < 0.001 |  | **🗸** |
| Mini mental state examination score < 27 | 462 (14.7%) | 213 | 55 (3.2%) | 407 (29%) | < 0.001 | **🗸** | **🗸** |
| Previous or actual smoking habit | 465 (14.3%) | 102 | 334 (18.9%) | 131 (8.7%) | < 0.001 |  |  |
| Inability to prepare and take medicines alone | 451 (13.7%) | 77 | 44 (2.5%) | 407 (26.4%) | < 0.001 | **🗸** | **🗸** |
| Chronic colitis | 425 (12.6%) | 0 | 125 (7%) | 300 (19%) | < 0.001 |  | **🗸** |
| Osteoarthritis | 425 (12.6%) | 0 | 197 (11.1%) | 228 (14.4%) | 0.003 |  |  |
| Inability to prepare food alone | 417 (12.5%) | 29 | 32 (1.8%) | 385 (24.7%) | < 0.001 | **🗸** | **🗸** |
| Heart murmur at physical examination | 404 (12.2%) | 52 | 94 (5.3%) | 310 (20%) | < 0.001 |  |  |
| Inability to use means of transportation alone | 402 (12.1%) | 46 | 31 (1.8%) | 371 (24%) | < 0.001 | **🗸** | **🗸** |
| Anaemia | 405 (12%) | 0 | 76 (4.3%) | 329 (20.8%) | < 0.001 | **🗸** | **🗸** |
| Reporting being sad for most of the time | 392 (11.9%) | 81 | 150 (8.5%) | 242 (16%) | < 0.001 |  |  |
| Obesity | 396 (11.8%) | 0 | 273 (15.3%) | 123 (7.8%) | < 0.001 |  |  |
| Reporting difficulties in making decisions | 394 (11.8%) | 12 | 81 (4.6%) | 313 (19.9%) | < 0.001 |  |  |
| Deafness and other hearing loss | 386 (11.5%) | 0 | 57 (3.2%) | 329 (20.8%) | < 0.001 | **🗸** | **🗸** |
| Reporting loss of appetite | 354 (10.9%) | 124 | 80 (4.5%) | 274 (18.6%) | < 0.001 | **🗸** | **🗸** |
| Heart rhythm alterations (at physical examination) | 358 (10.8%) | 39 | 89 (5%) | 269 (17.3%) | < 0.001 |  |  |
| Reporting suicidal ideas | 342 (10.7%) | 173 | 98 (5.6%) | 244 (17.1%) | < 0.001 |  |  |
| Heart failure | 353 (10.5%) | 0 | 43 (2.4%) | 310 (19.6%) | < 0.001 | **🗸** | **🗸** |
| Thyroid diseases | 352 (10.5%) | 0 | 153 (8.6%) | 199 (12.6%) | < 0.001 |  | **🗸** |
| Inability to perform house chores alone | 344 (10.3%) | 27 | 30 (1.7%) | 314 (20.1%) | < 0.001 |  | **🗸** |
| Reporting feeling worthless most of the time | 325 (10.2%) | 186 | 131 (7.4%) | 194 (13.7%) | < 0.001 |  | **🗸** |
| Reporting to have experienced fatigue in the last three months | 331 (9.8%) | 0 | 131 (7.4%) | 200 (12.7%) | < 0.001 |  |  |
| Atrial Fibrillation | 324 (9.6%) | 0 | 71 (4%) | 253 (16%) | < 0.001 | **🗸** | **🗸** |
| Dementia | 322 (9.6%) | 0 | 24 (1.3%) | 298 (18.8%) | < 0.001 | **🗸** | **🗸** |
| Depression and other mood disorders | 310 (9.2%) | 0 | 153 (8.6%) | 157 (9.9%) | 0.179 |  | **🗸** |
| Solid neoplasms | 299 (8.9%) | 0 | 125 (7%) | 174 (11%) | < 0.001 | **🗸** | **🗸** |
| Diabetes | 296 (8.8%) | 0 | 148 (8.3%) | 148 (9.4%) | 0.281 |  | **🗸** |
| Inability to manage finances alone | 272 (8.2%) | 40 | 24 (1.4%) | 248 (16%) | < 0.001 | **🗸** | **🗸** |
| Cerebrovascular diseases | 265 (7.9%) | 0 | 63 (3.5%) | 202 (12.8%) | < 0.001 | **🗸** | **🗸** |
| Slowness in movement (at physical examination) | 236 (7.2%) | 75 | 61 (3.4%) | 175 (11.5%) | < 0.001 |  |  |
| Inability to wash him/herself alone | 239 (7.1%) | 12 | 11 (0.6%) | 228 (14.5%) | < 0.001 | **🗸** | **🗸** |
| Reporting difficulties in concentration | 214 (6.8%) | 215 | 62 (3.5%) | 152 (10.9%) | < 0.001 |  |  |
| Osteoporosis | 228 (6.8%) | 0 | 58 (3.3%) | 170 (10.8%) | < 0.001 |  | **🗸** |
| Other muskulo-skeletal conditions | 222 (6.6%) | 0 | 71 (4%) | 151 (9.6%) | < 0.001 |  |  |
| Dorsopathies | 216 (6.4%) | 0 | 103 (5.8%) | 113 (7.1%) | 0.106 |  |  |
| Abnormal muscle tonus (at physical examination) | 207 (6.3%) | 65 | 28 (1.6%) | 179 (11.7%) | < 0.001 |  |  |
| Asthma | 205 (6.1%) | 0 | 113 (6.3%) | 92 (5.8%) | 0.528 |  |  |
| Swollen legs (at physical examination) | 202 (6%) | 24 | 38 (2.1%) | 164 (10.5%) | < 0.001 |  | **🗸** |
| Tremor (at physical examination) | 194 (5.9%) | 101 | 59 (3.4%) | 135 (8.9%) | < 0.001 |  |  |
| Living in a nursing home | 191 (5.7%) | 0 | 11 (0.6%) | 180 (11.4%) | < 0.001 | **🗸** |  |
| Glaucoma | 189 (5.6%) | 0 | 38 (2.1%) | 151 (9.6%) | < 0.001 |  |  |
| Cataract and other lens disorders | 184 (5.5%) | 0 | 37 (2.1%) | 147 (9.3%) | < 0.001 |  |  |
| Chronic obstructive pulmonary disease | 167 (5%) | 0 | 69 (3.9%) | 98 (6.2%) | 0.002 | **🗸** | **🗸** |
| Other eye diseases | 167 (5%) | 0 | 43 (2.4%) | 124 (7.8%) | < 0.001 | **🗸** |  |
| Using a wheelchair | 163 (4.9%) | 28 | 21 (1.2%) | 142 (9.1%) | < 0.001 |  |  |
| Inability to use the toilet alone | 161 (4.8%) | 14 | 10 (0.6%) | 151 (9.6%) | < 0.001 | **🗸** | **🗸** |
| Autoimmune disorders | 152 (4.5%) | 0 | 54 (3%) | 98 (6.2%) | < 0.001 |  |  |
| Incontinence | 149 (4.5%) | 63 | 18 (1%) | 131 (8.6%) | < 0.001 |  | **🗸** |
| Inability to dress alone | 150 (4.5%) | 17 | 10 (0.6%) | 140 (8.9%) | < 0.001 | **🗸** | **🗸** |
| Oesophageal, gastric or duodenal disorders | 146 (4.3%) | 0 | 65 (3.6%) | 81 (5.1%) | 0.036 |  |  |
| Blindness and other visual loss problems | 144 (4.3%) | 0 | 14 (0.8%) | 130 (8.2%) | < 0.001 | **🗸** | **🗸** |
| Inability to feed him/herself alone | 142 (4.2%) | 12 | 13 (0.7%) | 129 (8.2%) | < 0.001 | **🗸** | **🗸** |
| Inability to transfer from bed to chair alone | 140 (4.2%) | 10 | 9 (0.5%) | 131 (8.3%) | < 0.001 | **🗸** | **🗸** |
| Inflammatory arthropathy | 136 (4%) | 0 | 56 (3.1%) | 80 (5.1%) | 0.005 |  |  |
| Abnormal balance with eye closed (Romberg's test - at physical examination) | 109 (3.6%) | 346 | 17 (1%) | 92 (7.2%) | < 0.001 |  |  |
| Other cardiovascular disorder | 116 (3.4%) | 0 | 34 (1.9%) | 82 (5.2%) | < 0.001 | **🗸** |  |
| Reporting dyspnoea (at physical examination) | 109 (3.3%) | 20 | 22 (1.2%) | 87 (5.6%) | < 0.001 |  | **🗸** |
| Somatic stress disorder | 105 (3.1%) | 0 | 50 (2.8%) | 55 (3.5%) | 0.263 |  |  |
| Abnormal finger-nose test (at physical examination) | 96 (3%) | 214 | 20 (1.1%) | 76 (5.4%) | < 0.001 |  |  |
| Body mass index lower than 18 | 89 (2.9%) | 329 | 16 (0.9%) | 73 (5.7%) | < 0.001 |  |  |
| Agitation (at physical examination) | 96 (2.9%) | 76 | 30 (1.7%) | 66 (4.3%) | < 0.001 |  |  |
| Other genitourinary disorders | 85 (2.5%) | 0 | 31 (1.7%) | 54 (3.4%) | 0.002 |  |  |
| Cardiac valve disorders | 83 (2.5%) | 0 | 25 (1.4%) | 58 (3.7%) | < 0.001 | **🗸** |  |
| Chronic headache, migraine and facial nerve pain | 82 (2.4%) | 0 | 48 (2.7%) | 34 (2.2%) | 0.308 |  |  |
| Carotidal bruits (at physical examination) | 77 (2.3%) | 73 | 18 (1%) | 59 (3.8%) | < 0.001 |  |  |
| Other psychiatric disorders | 74 (2.2%) | 0 | 28 (1.6%) | 46 (2.9%) | 0.008 | **🗸** |  |
| Sleep disorders | 70 (2.1%) | 0 | 39 (2.2%) | 31 (2%) | 0.644 |  | **🗸** |
| Other neurological disorders | 65 (1.9%) | 0 | 28 (1.6%) | 37 (2.3%) | 0.106 | **🗸** |  |
| Bradycardias and other cardiac conduction disorders | 62 (1.8%) | 0 | 14 (0.8%) | 48 (3%) | < 0.001 | **🗸** |  |
| Inability to use the telephone alone | 55 (1.7%) | 55 | 5 (0.3%) | 50 (3.3%) | < 0.001 |  | **🗸** |
| Dysarthria (at physical examination) | 54 (1.6%) | 82 | 14 (0.8%) | 40 (2.6%) | < 0.001 |  |  |
| Allergy | 54 (1.6%) | 0 | 39 (2.2%) | 15 (0.9%) | 0.004 |  |  |
| Peripheral vascular disease | 54 (1.6%) | 0 | 19 (1.1%) | 35 (2.2%) | 0.008 | **🗸** | **🗸** |
| Chronic diseases of pancreas and gallbladder | 53 (1.6%) | 0 | 32 (1.8%) | 21 (1.3%) | 0.277 | **🗸** |  |
| Other metabolic disorders | 51 (1.5%) | 0 | 21 (1.2%) | 30 (1.9%) | 0.089 |  |  |
| Paresis (at physical examination) | 49 (1.5%) | 61 | 18 (1%) | 31 (2%) | 0.017 |  |  |
| Peripheral neural diseases | 49 (1.5%) | 0 | 18 (1%) | 31 (2%) | 0.022 |  | **🗸** |
| Disinhibited behaviour (physical exam) | 46 (1.4%) | 90 | 16 (0.9%) | 30 (2%) | 0.008 |  |  |
| Parkinson's disease and parkinsonism | 40 (1.2%) | 0 | 12 (0.7%) | 28 (1.8%) | 0.003 | **🗸** | **🗸** |
| Other respiratory disorders | 37 (1.1%) | 0 | 17 (1%) | 20 (1.3%) | 0.388 | **🗸** |  |
| Dysphagia | 36 (1.1%) | 82 | 11 (0.6%) | 25 (1.7%) | 0.005 |  |  |
| Losing at least 1 kg in the last 3 months | 33 (1%) | 19 | 14 (0.8%) | 19 (1.2%) | 0.214 |  |  |

Data were obtained from the complete dataset. P values are derived from chi-squared test for the difference of prevalence between younger and older subsamples.
